# Aging-Resistant Functionalized LDH–SAS/Nitrile-Butadiene Rubber Composites: Preparation and Study of Aging Kinetics/Anti-Aging Mechanism

**DOI:** 10.3390/ma11050836

**Published:** 2018-05-18

**Authors:** Tianxiang Li, Zhengren Shi, Xianru He, Ping Jiang, Xiaobin Lu, Rui Zhang, Xin Wang

**Affiliations:** 1 School of Materials Science and Engineering, Southwest Petroleum University, 8 Xindu Avenue, Chengdu 610500, Sichuan, China; 201521000651@stu.swpu.edu.cn (T.L.); qianyege@163.com (Z.S.); 201521000645@stu.swpu.edu.cn (P.J.); xblu1009@163.com (X.L.); 2 Institut für Physik, Universität Rostock, Albert-Einstein-Str. 23-24, 18051 Rostock, Germany

**Keywords:** layered double hydroxides (LDHs), nitrile-butadiene rubber (NBR), 4-Amino-benzenesulfonic acid monosodium salt (SAS), thermal oxidative aging resistance properties

## Abstract

With the aim of improving the anti-aging properties of nitrile-butadiene rubber (NBR), a functional organic filler, namely LDH–SAS, prepared by intercalating 4-amino-benzenesulfonic acid monosodium salt (SAS) into layered double hydroxides (LDHs) through anion exchange, was added to nitrile-butadiene rubber (NBR), giving the NBR/LDH–SAS composites. Successful preparation of LDH–SAS was confirmed by XRD, TGA and FTIR. LDH–SAS was well dispersed in the NBR matrix, owing to its strong interaction with the nitrile group of NBR. The obtained NBR/LDH–SAS composites exhibited excellent thermo-oxidative aging resistance as shown by TGA-DSC. Further investigation by ATR-FTIR indicated that SAS can capture the radical groups, even during the aging process, which largely accounts for the improved aging resistance.

## 1. Introduction

Nitrile-butadiene rubber (NBR) is a synthetic rubber widely used for various applications, such as seals in the aerospace, automotive and oil petroleum industries [1,2,3]. Compared with most commercial rubbers, NBR has beneficial properties including excellent solvent resistance, bonding performance, and a wide temperature application range [4]. In the petroleum industry, NBR plays a key role in sealing drill devices [5,6]. However, the NBR backbone contains a relatively large number of double bonds, even after vulcanization, which are prone to thermo-oxidative degradation. This is detrimental to the exploitation of ultra-deep petroleum wells, where seals are required to be resistant to thermo-oxidative aging [7]. For this reason, hydrogenated nitrile butadiene rubber (HNBR) is used to replace NBR in the seals [8,9,10], although its cost is much higher than that of NBR. Moreover, hydrogenation itself is a high energy-consuming procedure, which further increases the cost of the products as well as adding to environmental problems. A convenient, economical and environmentally friendly method is desirable.

To overcome the cost limitations for industrial applications, filler modification is an effective method for improving the anti-aging properties of bulk polymers [7,11]. Fillers like montmorillonite, silicon dioxide, graphite oxide and Fe_2_O_3_ have been widely reported to improve mechanical properties, flame retardancy and thermal stability of bulk polymers [12,13,14,15]. To date, layered clay minerals are among the most investigated inorganic fillers due to their high surface-to-volume ratio [16] and reasonable cost [17]. The layered fillers have also been reported to improve the thermal stability of polymers [18,19,20]. Studies have shown that a tortuous structure forms between the layered filler and the rubber matrix, which reduces oxygen diffusion and thereby decreases the rate of thermo-oxidative aging [19]. However, these inorganic fillers also have drawbacks, attributed to weak interaction forces (e.g., van der Waals) between the matrix and filler. Thus, phase separation can easily occur between these two components. Chemical methods, like surface functionalization and hybrid and organic intercalation, can enhance these interactions [7,21,22,23,24,25,26,27].

In this context, layered double hydroxides (LDHs), also known as anionic clays, have received attention in recent years as a functional filler to improve mechanical, flame-retardant and thermal stability properties of polymers [28,29,30,31,32]. In contrast to other clay minerals with negatively charged layers, LDHs contain positively charged mixed metal hydroxide layers separated by charge-balancing anions and water molecules [4]. It is difficult for the polymer chains to intercalate into the LDH interlayers due to the small interlayer gallery space [33] and the hydrophilic nature of the hydroxide sheets. In addition, there may be weak interactions between the LDHs and polymers. To address these problems, the original interlayer inorganic anions (CO_3_^2−^, NO_3_^−^ and Cl^−^) in LDHs can be replaced by organic anions (styrene sulfonate, sodium dodecylsulfate, sodium dodecyl benzenesulfonate or sodium stearate), thus increasing the gallery height of the LDHs [34].

Many polymers, including polyethylene [35], polyvinyl chloride [36,37] and polystyrene [38], have been modified with LDHs to prepare composites and improve their thermal stability and flammability performance. In addition, organically modified LDHs can also be applied to rubber composite materials [39]. NBR has similarly been modified with LDHs to improve its mechanical, thermal and optical properties [32,40,41,42,43,44,45], with just previous one report on the influence of LDHs on the thermal oxidative aging resistance in NBR materials existing in the literature [7]. NBR complexes using modified LDHs have also been prepared to improve mechanical properties. For example, the influence of sodium lignosulfonate-modified LDHs (SLS-LDH) and sodium styrene sulfonate-modified LDHs (LDH-SSS) on the mechanical properties of NBR has been investigated [41,43]. In our previous study [7], double bond-containing organic fillers LDH-SSS (sodium styrene sulfonate modified LDHs) were successfully prepared and used in NBR composites. A grafting reaction between the organic filler and NBR molecular chain was found to increase the saturation of NBR molecular chains, which reduced chain scission during aging. However, a drawback in this work is that the grafting reaction is hard to control and the LDHs are easily over grafted, which might lead to compromised anti-aging performance of the filler. For this reason, a different method is desirable to provide a more controllable and stable route for LDHs to function to improve the anti-aging properties of NBR.

In this study, mechanical blending replaces the chemical grafting reaction in the preparation of LDHs modified by 4-amino-benzenesulfonic acid monosodium salt (SAS) and blended with NBR (NBR/LDH–SAS). As a control sample, an unmodified LDHs–NO_3_ precursor was blended with NBR (NBR/LDHs–NO_3_). A detailed analysis of these composites is given to show their structure, morphology, mechanical properties, thermal properties and the aging mechanism, in comparison with the unmodified NBR and NBR/LDH–NO_3_ composites. Using organic-functionalized LDHs as stabilizers to modify NBR is a promising method for reducing the use of monomer stabilizers, which can reduce environmental pollution in industry applications. This work sets the stage for future work on preparing rubber composites with high anti-aging ability and more environmentally friendly characteristics.

## 2. Experimental

### 2.1. Materials

Nitrile-butadiene rubber with 29 wt % of acrylonitrile was obtained from China Lanzhou Petrochemical Corporation (Lanzhou, China). Magnesium nitrate (Mg(NO_3_)_2_·6H_2_O), aluminum nitrate (Al(NO_3_)_3_·9H_2_O), NaOH, zinc oxide, sulfur, stearic acid and dioctyl phthalate (DOP) were supplied by China Kelong Chemical Reagent Factory (Chengdu, China). 4-Amino-benzenesulfonic acid monosodium salt (SAS), *N*-phenyl-2-naphthylamine (antioxidant D) and 2,2′-Dibenzothiazole disulfide (DM) were purchased from China Aladdin Industrial Corporation (Shanghai, China). All materials were used as received without further purification unless otherwise indicated.

### 2.2. Synthesis of LDH–NO_3_

Two-dimensional LDH–NO_3_ precursors were prepared by following a typical coprecipitation procedure [46]. First, a 2.0 mol/L NaOH solution was prepared by dissolving 10 g of NaOH in 400 mL of deionized water. Nitrate solution was prepared by dissolving 19.2 g of Mg(NO_3_)_2_·6H_2_O (0.075 mol) and 9.37 g of Al(NO_3_)_3_·9H_2_O (0.025 mol) in 400 mL of deionized water. Second, 400 mL of NaOH solution was added dropwise into the nitrate solution at 25 °C. The pH value of the mixture solution was adjusted to 10. Then, the well mixed solution was crystallized at 85 °C for 24 h under nitrogen atmosphere. The resultant white precipitate was filtered and rinsed with deionized water to remove the unreacted ions, followed by drying in a vacuum oven at 80 °C for 12 h.

### 2.3. Preparation of LDH-SAS by an Organic Anion

LDH–SAS was prepared by an anion exchange method. Ten grams of LDH–NO_3_ precursor and 200 mL of deionized water were added into a three-necked bottle. The mixture was heated to 100 °C and stirred for 6 h until the LDH–NO_3_ precursor was fully swollen in deionized water, after which the temperature was decreased from 100 to 85 °C. A desired amount of SAS solution was added into the mixture, and then HNO_3_ solution was added dropwise to obtain a pH value of 10. All the operations were performed under nitrogen atmosphere. Finally, the resultant light brown precipitate was filtered and washed with deionized water to remove the unreacted ions. The solid obtained was dried in a vacuum oven at 80 °C for 12 h.

### 2.4. Preparation of NBR/LDH Composites

NBR, additives and LDHs were mixed in a two-roll mixing mill. The components of the NBR composites are given in Table 1. The temperature of the two-roll was maintained at 30–40 °C via circulating water. The rubber composites were vulcanized in a press vulcanizer under a pressure of 10 MPa at 150 °C for t_90_. The t_90_ of each rubber composite is shown in Table 2. The vulcanized rubber composites were cut into dumbbell-shaped specimens or other shapes for particular measurements.

### 2.5. Aging Test of NBR

Thermo-oxidative aging experiments were carried out in a Mingzhu 401-A aging test chamber (Mingzhu Test Machinery Co., Ltd., Yangzhou, China) at 90 °C for 96 h. The NBR sample was cut into a dumbbell shape with thickness of 2 mm, width of 6 mm and the middle standard length of 25 mm.

### 2.6. Characterization and Measurements

X-ray diffraction (XRD) diffractograms of the LDHs and rubber specimens were obtained using a PANalytical B.V. X Pert PRO MPD X-ray diffractometer (Almelo, The Netherlands), equipped with Cu Kα radiation (λ = 0.154 nm). The scanning diffraction angle was from 3 to 70° with a scanning rate of 4°/min.

Fourier transform infrared spectra (FTIR) of LDHs were recorded using a Thermo Fisher Scientific Nicolet iS50 infrared spectrometer (Waltham, MA, USA). The samples were pre-dried and pressed with KBr powder into pellets. Attenuated total reflectance Fourier transform infrared spectra (ATR-FTIR) of NBR composites were also recorded on the same device. The scanning wavenumbers ranged from 4000 to 500 cm^−1^.

Thermogravimetric analysis (TGA) of dried LDHs was done on a Mettler Toledo TGA/SDTA85 thermal analyzer (Zurich, Switzerland) under nitrogen atmosphere with a flow rate of 20 mL/min. The heating rate was 10 °C/min from 25 to 800 °C. Thermogravimetric-Differential Scanning Calorimeter synchronization analysis (TGA-DSC) of unmodified NBR and NBR composites was carried out on a NETZSCH STA449F3 Jupiter instrument (Bavaria, Germany)with heating rates varying from 5 to 20 °C/min, from 40 to 800 °C. The measurements were done under oxygen atmosphere with a gas flow rate of 20 mL/min.

Tensile tests of NBR composites were carried out using a MTS CMT 6104 universal tensile testing machine (MTS Systems (China) Co., Ltd., Shanghai, China) at 25 °C by following a Chinese national test standard of GB/T528-1998. The cross-head speed was 500 mm/min during the tensile test. Each sample was tested five times and the average values were taken. The standard deviations (SD) of these data were obtained with Equation (1) [47]:

(1) $$\mathrm{SD}=\sqrt{\frac{\sum_{i=1}^{n}{\left(x_{i}-\bar{x}\right)}^{2}\alpha }{n-1}},$$

where n is the total number of experiments, $x_{i}$ are the experimental values, and $\bar{x}$ is the average of the experimental values.

Dynamic mechanical analysis (DMA) of NBR specimens was performed on a TA Q800 DMA (TA Instruments, New Castle, DE, USA) in double cantilever beam mode with a frequency of 1 Hz from −50 to 20 °C at a heating rate of 3 °C/min.

Scanning electron microscopy (SEM) analysis was performed on a JSM-6360LV instrument (Japan Electronics Co., Tokyo, Japan) to study the surface morphology of the LDH particles and the fracture surface morphology of composite specimens.

## 3. Results and Discussion

### 3.1. Structural and Chemical Properties of LDHs

**Figure 1:**
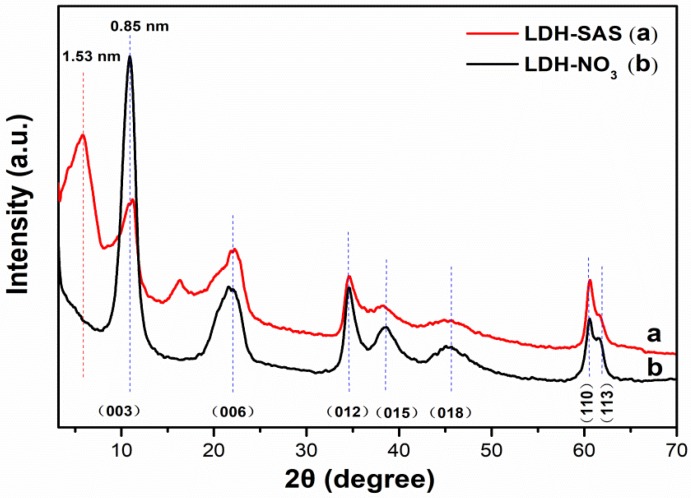
X-ray diffractograms of (**a**) LDH-SAS and (**b**) LDH-NO_3_. The d-spacings of the samples are marked at the corresponding peaks.

In general, the layer structure of LDHs can be obtained from their X-ray diffractogram. The existence of crystal planes at (003), (006), (012), (015), (018), (110) and (113) indicated that the prepared LDH materials were highly crystalline [38], as shown in Figure 1. The interlayer distance of the LDHs was calculated through Bragg’s equation. For LDH–SAS, the (003) diffraction peak at 5.84°giave an interlayer distance of 1.53 nm, which was larger than that for LDH–NO_3_ at 0.85 nm. This indicates that the SAS molecules were successfully intercalated into the LDH interlayer through anion exchange [48]. However, a relatively weak diffraction peak at 10.36° was also observed for LDH–SAS in Figure 1, indicating the existence of a small amount of LDH–NO_3_ in LDH–SAS [16]. The diffraction peaks of LDH–NO_3_ were all narrower and sharper than those of LDH–SAS, indicating that the crystallinity of LDH–NO3 was higher than that of LDH–SAS. This may have been caused by the expanded interlayer spacing and might disturb the crystal structure of LDH–SAS.

**Figure 2:**
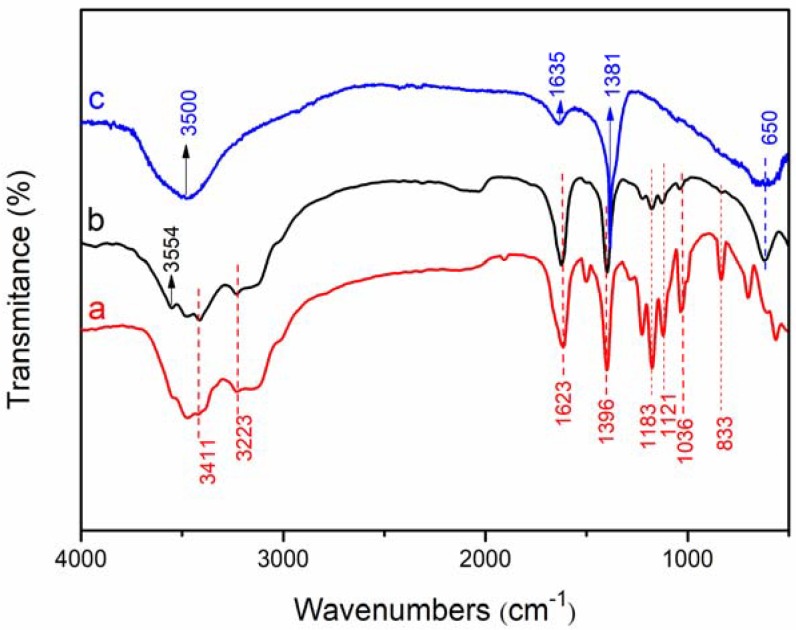
FT-IR spectra of (**a**) pure SAS, (**b**) LDH–SAS and (**c**) LDH–NO_3._

The interaction between LDHs and SAS is important for explaining the successful intercalation of LDH-SAS, which could be studied through FTIR spectroscopy [16]. As shown in Figure 2, both LDH–SAS and LDH–NO_3_ have absorption bands at 3500–3400 cm^−1^, 700–600 cm^−1^ and about 1385 cm^−1^. These typical LDH absorption bands are attributed to the stretching vibration of hydroxyl groups [43], the bending vibration of metal hydroxide sheets [49] and the stretching vibration of NO_3_^−^ in the LDH interlayer [41,49], respectively. The stretching bands at 1623 and 1635 cm^−1^ are attributed to the bending vibration of the LDH interlayer–water interaction [16,37,41]. It should be noticed that the interlayer–water absorbing peak shifted from 1635 to 1623 cm^−1^ when LDH–NO_3_ was changed to LDH–SAS. This may have been caused by the presence of hydrogen bonding between the interlayer–water and the sulfonate group of SAS, as was found in a similar system [7]. The LDH–SAS band had a new absorption peak at 3554 cm^−1^, because the intercalation of SAS into LDHs caused relaxation of the interlayer structure, which allowed more free water molecules to enter the interlayers. For LDH–SAS, the absorption peaks at about 3411 and 3223 cm^−1^ were mainly related to the primary amine bending vibration of N–H. The peaks at about 1183, 1122 and 1036 cm^−1^ of LDH–SAS are attributed to the stretching vibration of sulfonate groups [41], and the peak at about 833 cm^−1^ is the bending vibration of 1,4-substituted benzene rings. X-ray diffraction and FTIR spectroscopy thus indicated the successful preparation of LDH–SAS.

### 3.2. Thermal Stability of LDHs

**Figure 3:**
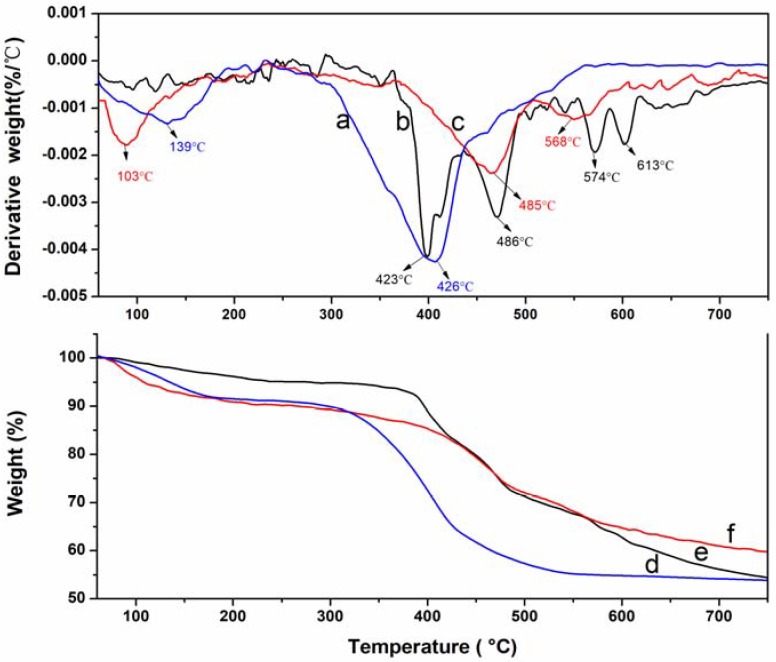
Differential thermogravimetric analysis (DTG) thermograms of (**a**) LDH–NO_3_, (**b**) pure SAS, (**c**) LDH–SAS and thermogravimetric analysis (TGA) thermograms of (**d**) LDH–NO_3_, (**e**) pure SAS, (**f**) LDH–SAS.

LDH–NO_3_ had two obvious weight loss stages, as shown in Figure 3 (curves a, d). The first stage occurred at about 150 °C (termed “low-temperature decomposition”). This is attributed to the loss of free water and interlayer water molecules. The second stage (termed “high-temperature decomposition”) occurred at about 420 °C, and was caused by degradation of the hydroxide layer of LDH–NO_3_.

LDH–SAS had a smoother thermal degradation curve and wider thermal degradation temperature range than LDH–NO_3_, as shown in Figure 3 (curves c, e). Its weight loss curve showed three main stages. The low temperature decomposition stage (the loss of physical free water and interlayer water molecules) began at a lower temperature (103 °C) compared to that of LDH–NO_3_ (139 °C). This can be explained, on one hand, by the increased interlayer spacing and relaxation of its structure caused by SAS intercalation, which allows easier loss of the interlayer water, and, on the other hand, because the interactions between interlayer water molecules of LDH–NO_3_ and interlayer nitrate ions are stronger than those between interlayer water molecules and SAS ions. [32]. The second decomposition stage, due to hydroxide layer degradation, occurred at about 470 °C, higher by about 50 °C than for LDH–NO_3_. A third stage took place at about 570 °C, and was caused by the degradation of SAS in the interlayer, as supported by the SAS curves shown in Figure 3 (curves b, f). This shows that LDH–SAS has greater thermal stability and a longer retention time during thermal degradation than LDH–NO_3_ [50].

### 3.3. XRD Analysis of NBR Composites

**Figure 4:**
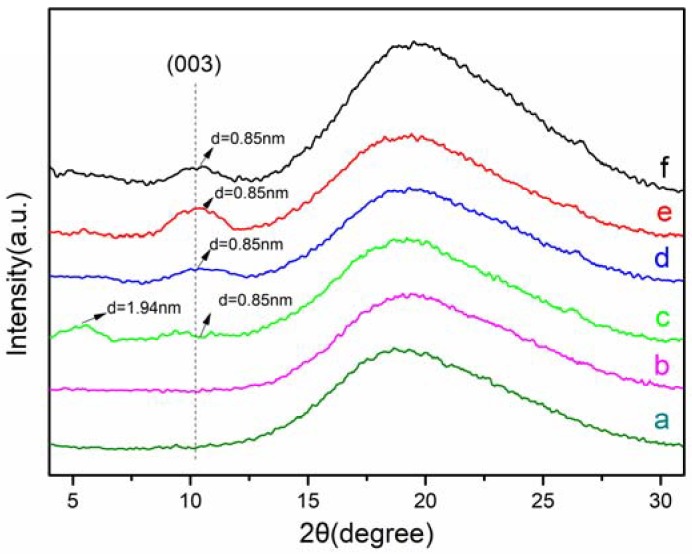
X-ray diffractograms of the NBR composites: (**a**) unmodified NBR before aging, (**b**) unmodified NBR after aging, (**c**) NBR/LDH–SAS before aging, (**d**) NBR/LDH–SAS after aging, (**e**) NBR/LDH–NO_3_ before aging, (**f**) NBR/ LDH–NO_3_ after aging.

XRD was used to study the exfoliation behavior of LDHs in the rubber. The X-ray diffractograms of the unmodified NBR, both before and after aging, shown in Figure 4a,b have only one broad peak at about 19°, attributed to the amorphous region of NBR. When LDH–SAS was added to the rubber, the basal spacing (d_003_) increased from 1.53 to 1.94 nm (Figure 1a and Figure 4c), indicating that some NBR chains entered the interlayer of LDH–SAS during the mixing and vulcanization processes. This also indicates that LDH–SAS has a larger layer spacing and excellent compatibility with NBR molecular chains. For NBR/LDH–SAS after aging, however, the diffraction peak corresponding to the crystal face of (003) disappeared, as shown in Figure 4d. This could be explained by the fact that more NBR chains entered the LDH–SAS interlayer and formed cross-links during the aging process, making LDH-SAS completely exfoliate in NBR/LDH–SAS. In contrast, LDH–NO_3_ showed no change in its basal spacing (d_003_), which remained at about 0.85 nm after being added to NBR, as shown in Figure 1b and Figure 4e,f. This indicates that LDH–NO_3_ underwent no further exfoliation in the NBR matrix, probably because the smaller LDH–NO_3_ basal spacing prevented the NBR chain from moving into the LDH–NO_3_ interlayer.

### 3.4. Mechanical Properties of NBR Composites

**Figure 5:**
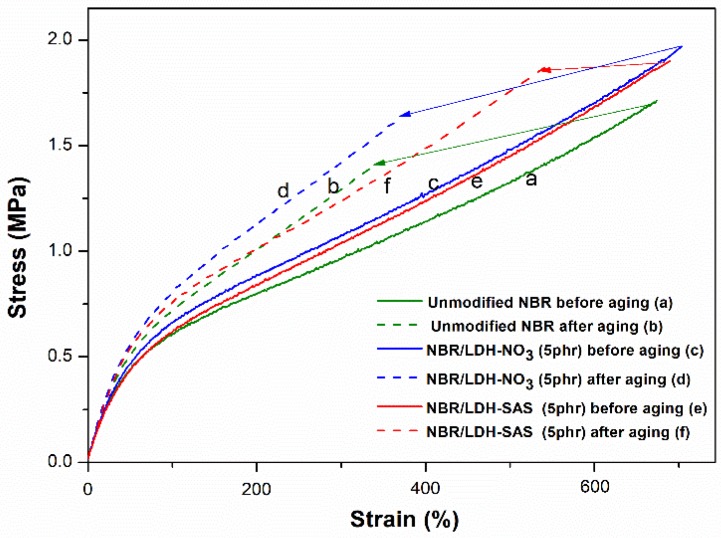
Stress–strain curve of different NBR composites (unmodified NBR, NBR with 5 phr LDH–NO_3_ and NBR with 5 phr LDH–SAS) before and after aging.

Tensile tests were conducted to evaluate the mechanical properties of the NBR/LDH composites. The selected stress–strain curves of different NBR composites are shown in Figure 5. The tensile strength and elongation at failure of all NBR composites after aging decreased. However, for NBR/LDH–SAS composites with 5 phr of LDH–SAS, both the retention of tensile strength and elongation at failure were higher than those of the unmodified NBR and NBR/LDH–NO_3_ composites with 5 phr of LDH–NO_3_.

**Figure 6:**
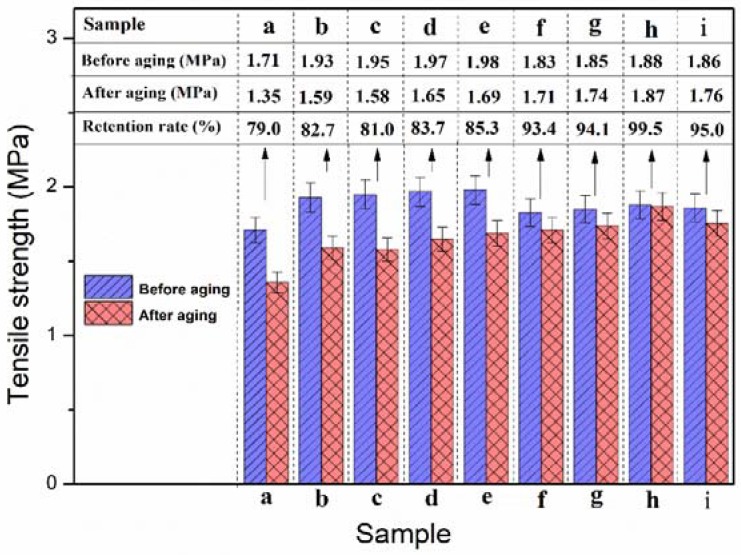
The tensile strength of NBR/LDHs composites before and after aging: (**a**) unmodified NBR, (**b**) NBR/LDH–NO_3_ with 1 phr, (**c**) NBR/LDH–NO_3_ with 3 phr, (**d**) NBR/LDH–NO_3_ with 5 phr, (**e**) NBR/LDH–NO_3_ with 7 phr, (**f**) NBR/LDH–SAS with 1 phr, (**g**) NBR/LDH–SAS with 3 phr, (**h**) NBR/LDH–SAS with 5 phr, (**i**) NBR/LDH–SAS with 7phr.

**Figure 7:**
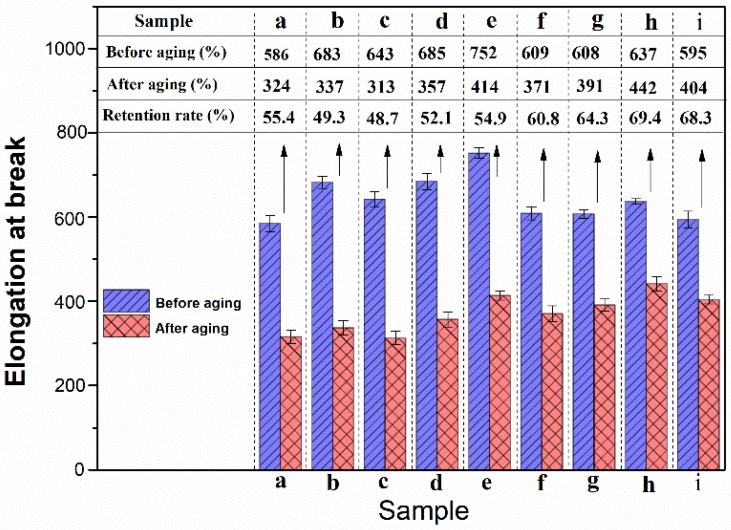
The elongation at break of NBR/LDHs composites before and after aging: (**a**) unmodified NBR, (**b**) NBR/LDH–NO_3_ with 1 phr, (**c**) NBR/LDH–NO_3_ with 3 phr, (**d**) NBR/LDH–NO_3_ with 5 phr, (**e**) NBR/LDH–NO_3_ with 7 phr, (**f**) NBR/LDH–SAS with 1 phr, (**g**) NBR/LDH–SAS with 3 phr, (**h**) NBR/LDH–SAS with 5 phr, (**i**) NBR/LDH–SAS with 7 phr.

The average tensile strength and elongation at break are shown in Figure 6 and Figure 7, respectively.

Both NBR/LDH–NO_3_ and NBR/LDH–SAS had a higher tensile strengths and elongation at break than the unmodified NBR. The LDH–NO_3_ was better than LDH–SAS at improving these properties; this might be because LDH–NO_3_ accelerates NBR vulcanization more [41]. However, LDH–SAS maintains a higher tensile strength and elongation at break than the unmodified NBR and NBR/LDH–NO_3_ after thermo-oxidative aging. In particular, the retention rates of tensile strength and elongation at break point of NBR/LDH–SAS (5 phr) composite were 99% and 70%, respectively.

### 3.5. Micromorphology of LDHs and Fractured Surface of NBR Composites

**Figure 8:**
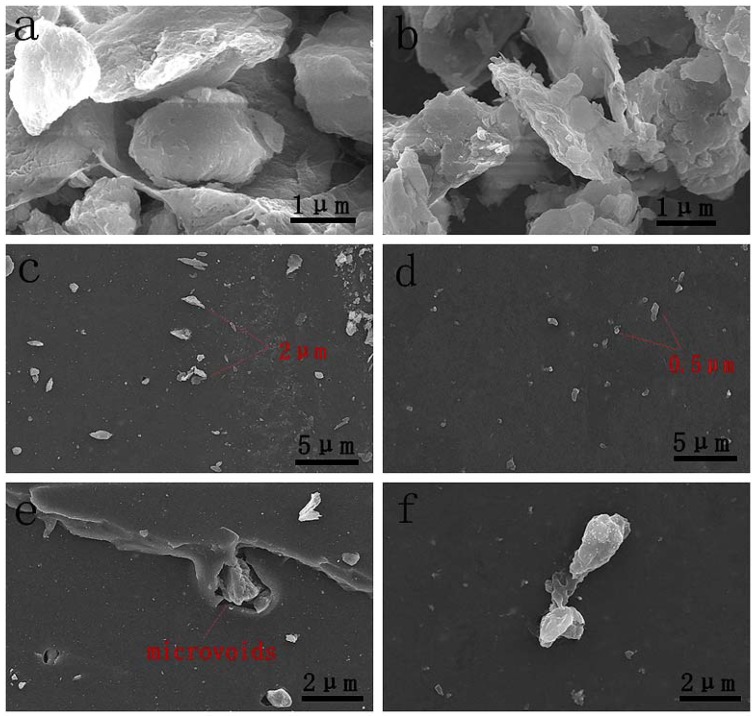
SEM images of LDH particles and fractured surfaces of NBR composites: (**a**) LDH–NO_3_ particles, (**b**) LDH–SAS particles, (**c**) NBR/LDH–NO_3_ before aging, (**d**) NBR/LDH–SAS before aging, (**e**) NBR/LDH–NO_3_ after aging, (**f**) NBR/LDH–SAS after aging.

The micromorphology of LDHs and the fractured surface micromorphology of NBR/LDH composites were studied by SEM, with representative images shown in Figure 8. Both the LDH–NO_3_ and LDH–SAS particles showed typical lamellar hexagons, with lateral dimensions of about 2–3 μm for LDH–NO_3_ and 1–2 μm for LDH–SAS. Compared with LDH–SAS, the LDH–NO_3_ particles had better crystal structures and more obvious hexagonal shapes. This is consistent with the XRD results in Figure 1, from which it was concluded that the intercalation of SAS in the LDH interlayers expanded the layer spacing and perturbed the crystal structure of LDH–SAS. Furthermore, LDH–NO_3_ aggregates in the rubber matrix reached sizes as large as 2 μm (Figure 8c), which might have been caused by the hydrophilicity and the large specific surface area as well as the strong filler–filler interactions of LDH–NO_3_ [51]. Compared to LDH–NO_3_, LDH–SAS had a better dispersion in the rubber matrix due to the stronger interactions of its organic groups with the NBR matrix.

As shown in Figure 8e, some microvoids were formed around the LDH–NO_3_ particles in NBR/LDH–NO_3_ composites after aging, which were possibly caused by the weak interaction between LDH–NO_3_ and the NBR matrix. Microvoids can decrease the mechanical strength of NBR/LDH–NO_3_ composites, which explains the results of the mechanical tests shown in Figure 6 and Figure 7. On the one hand, microvoids provide a channel to allow oxygen to penetrate NBR, enhancing thermo-oxidative processes. On the other hand, these microvoids can also increase the possibility of stress concentration. In contrast, no obvious microvoids were found in NBR/LDH–SAS after aging, as shown in Figure 8f. In line with the XRD result, this could be attributed to the organic–organic bonding layer between the intercalated NBR molecules and LDH–SAS that inhibits the formation of microvoids.

### 3.6. Dynamic Mechanical Analysis (DMA) of NBR Composites

**Figure 9:**
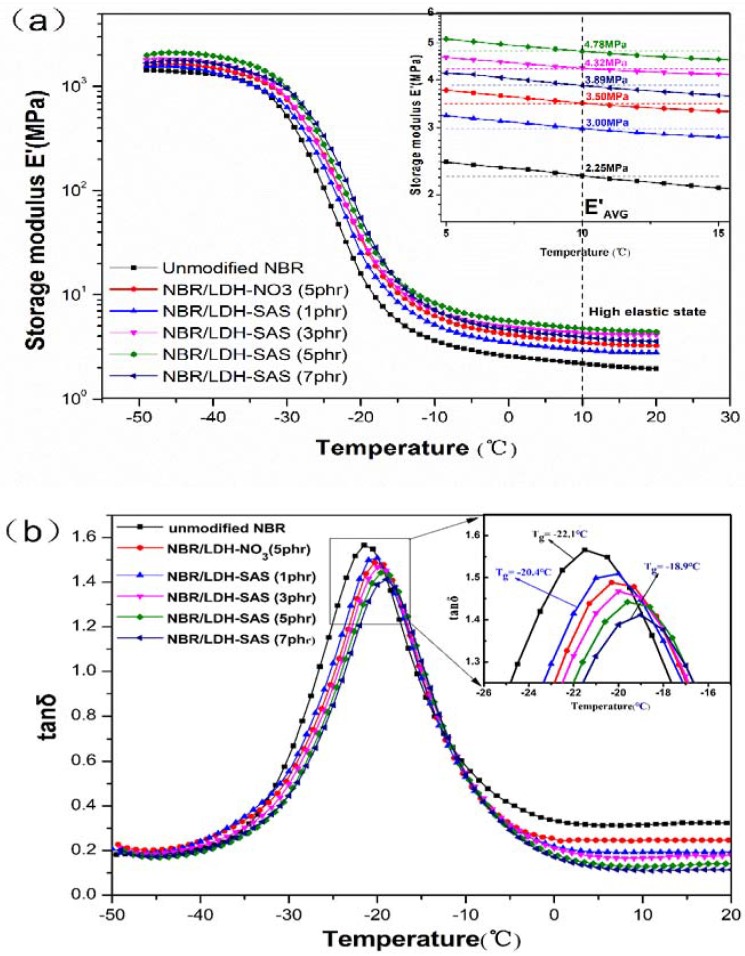
(**a**) Storage modulus and (**b**) tangent delta obtained from dynamic mechanical analysis of LDH-filled NBR composites. E′_AVG_ is average of storage modulus from 278.15 K to 288.15 K, which was used to represent the storage modulus of 283.15K.

The elasticity of a material can also be characterized by its storage modulus (E′) [34]. The NBR/LDH–SAS composites with 5 phr of LDH–SAS had the highest E′ among the samples studied, and all NBR/LDH-SAS composites had a higher E′ at 10 °C than unmodified NBR, as shown in Figure 9a. This might be caused by the high aspect ratio of LDH–SAS particles [52] and the interactions between NBR and LDH–SAS particles which limit the mobility of NBR molecules. However, for the NBR/LDH–SAS composite with 7 phr LDH–SAS, E′ was slightly decreased because of the agglomeration. It was observed that the E′ of NBR/LDH–SAS was higher than that of NBR/LDH–NO_3_ at the same 5 phr of filler loading, because LDH–SAS particles are easy to strip in the NBR matrix due to the larger layer spacing of LDH–SAS than that of LDH–NO_3_, as well as LDH–SAS having stronger interaction with NBR than LDH–NO_3_.

The glass transition temperature (Tg) determined from the tan δ peak for all NBR/LDH composites was approximately 2 °C higher than that of the unmodified NBR, as shown in Figure 9b, which is explained by the restriction of the mobility of NBR chains by LDHs [27,53,54,55,56,57,58]. Moreover, the Tg of NBR/LDH–SAS (5 phr) was higher than that of NBR/LDH–NO_3_ (5 phr), due to the organic ingredient (SAS) in LDH–SAS increasing the interactions between LDH–SAS and the NBR matrix. In addition, the insertion of NBR molecules into the interlayers of LDH–SAS further restricted the mobility of NBR [59]. Generally, the height of the tan δ peak reflects the energy damping characteristics of a material [34,60]. The tan δ peaks of NBR/LDH–SAS gradually decreased with increasing LDH–SAS content, indicating that the LDH–SAS increased the stiffness of the composites when well-dispersed in NBR [34,53]. In addition, the height of the tan δ peak of the NBR/LDH–SAS with 5 phr LDH–SAS was lower than that of NBR/LDH–NO_3_ with 5 phr LDH–NO_3_, because of the better dispersity of LDH–SAS compared to LDH–NO_3_ based on the results shown in Figure 8.

In order to further analyze the interaction between NBR and filler, the number of network chains per unit volume of rubber, defined as N_1_, was calculated from the state equation of cross-linked rubber (Equation (2)) [61] and the following equations:

(2) $${\sigma =3N}_{1}\mathrm{KT}\varepsilon ,$$

(3) σ=Eε,

(4) $${E=3N}_{1}\mathrm{KT},$$

where σ is the stress, K is the Boltzmann constant (K = 1.38 × 10^−23^ J/K), T is the absolute temperature, ε is the strain, and E is Young’s modulus. For rubber materials, the storage modulus (E′) in the elastic state is assumed to be approximately equal to its Young’s modulus (E). Therefore, Equation (4) can be rewritten as

(5) $${E^{\prime }=3N}_{1}\mathrm{KT}.$$

The E′ of NBR at 283.15 K (E′_AVG_) was chosen to calculate N_1_ and ΔN_1_ from Equation (5), and the results are listed in Table 3, where ΔN_1_ is the difference in the number of network chains between NBR/LDH composites and unmodified NBR.

**Scheme 1:**
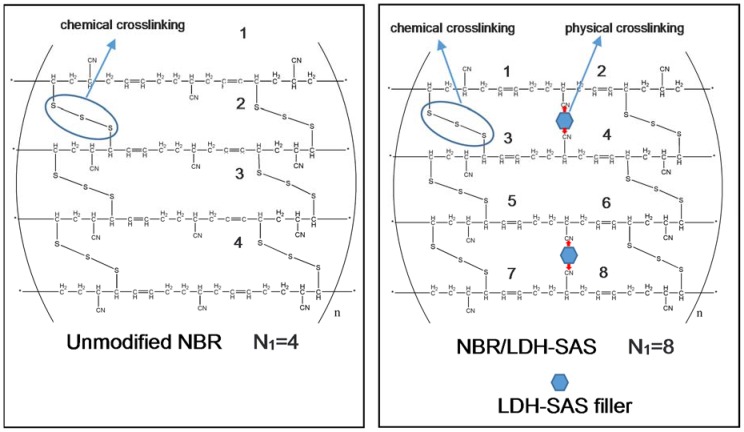
Schematic illustration of the increase of network chains per unit volume due to the formation of physical cross-linking points between LDH–SAS and NBR.

It can be seen from Table 2 that the N_1_ of the NBR composite after the addition of the filler was significantly larger than that of the unmodified NBR, indicating that there was a strong interaction between the fillers and the rubber molecules, as illustrated in Scheme 1. The ΔN_1_ of NBR/LDH–SAS (5 phr) was twice that of NBR/LDH–NO_3_ (5 phr), indicating that the SAS increased the interactions between LDH–SAS and the NBR molecular chain, resulting in more physical cross-linked sites. With an increase in LDH–SAS content, ΔN_1_ gradually increases at first, but begins to decrease when the LDH–SAS content surpasses 5 phr, which suggests that LDH–SAS aggregation reduces the physical cross-link points between the filler and rubber matrix.

### 3.7. Aging Mechanism of the NBR Composites

**Figure 10:**
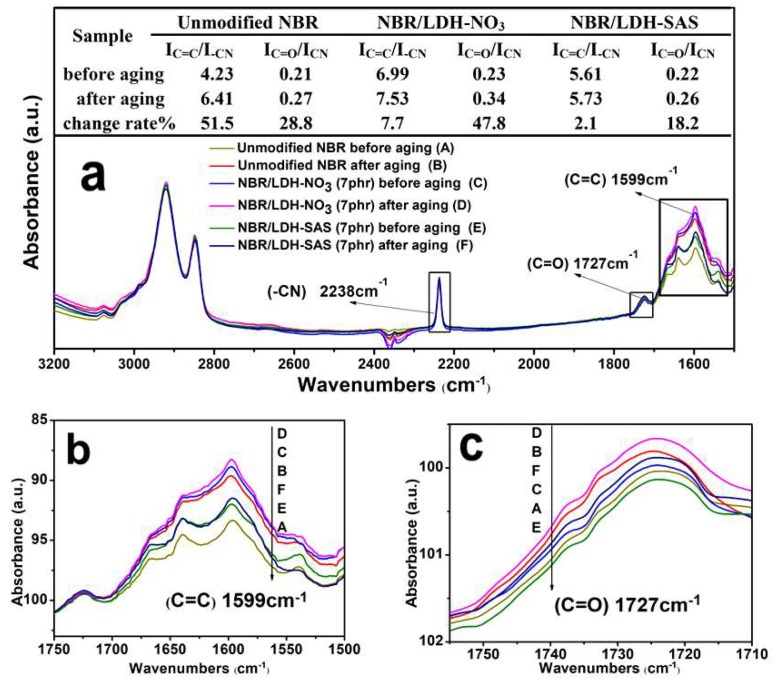
(**a**) FTIR-ATR spectra of NBR composites before and after aging, with the intensity of characteristic peaks at: (**b**) 1599 cm^−1^, and (**c**) 1727 cm^−1^.

In order to understand the aging mechanism of NBR/LDH composites, the changes in the chemical groups of NBR/LDHs before and after aging were analyzed using ATR-FTIR, as shown in Figure 10. The peaks at 1727 cm^−1^, 1599 cm^−1^ and 2238 cm^−1^ are attributed to the vibration of C=O, C=C and –CN, respectively [7,62]. NBR–CN was used as a reference to reflect the changes in other chemical groups, given that the –CN group is essentially stable during thermo-oxidative aging. I_C=O_/I_CN_ and I_C=C_/I_CN_ represent the peak intensities used to follow the chemical group changes [47].

**Figure 11:**
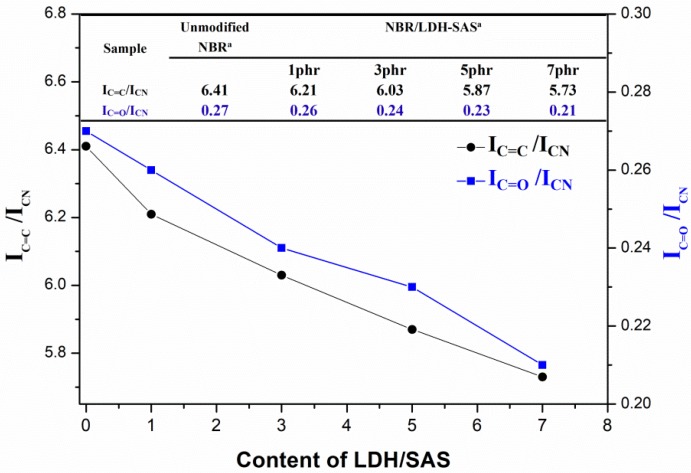
Evolution of the absorbance ratios (I_C=C_/I_CN_ and I_C=O_/I_CN_) of NBR composites with different LDH–SAS contents after aging.

Changes in the values of I_C=O_/I_CN_ and I_C=C_/I_CN_ are shown in Figure 10. The increase in I_C=C_/I_CN_ was caused by the scission of NBR main chains, and the increase of I_C=O_/I_CN_ was caused by the oxidation of NBR main chains [63]. The I_C=C_/I_CN_ and I_C=O_/I_CN_ values increased to different degrees after thermo-oxidative aging for the NBR composites, as shown in Figure 10a, indicating that both scission and oxidation of the NBR molecules occurred during thermo-oxidative aging [62]. For the unmodified NBR, the I_C=C_/I_CN_ and I_C=O_/I_CN_ increased by 51.5% and 28.8%, respectively, after aging (Figure 10a), indicating significant scission and oxidation during the aging process. However, for NBR/LDH–NO_3_, I_C=O_/I_CN_ and I_C=C_/I_CN_ increased by 47.8% and 7.7%, respectively, due to the accelerated effect of LDH–NO_3_ on the sulfur vulcanization [41] and the reduced content of unsaturated carbon–carbon double bonds in the composites. On the other hand, as discussed previously, the microvoids around the LDH–NO_3_ particles provided a channel for oxygen to react with NBR molecules and enhance the aging. For NBR/LDH–SAS, I_C=C_/I_CN_ and I_C=O_/I_CN_ changed by only 2.1% and 18.2%, respectively, which was attributed to the aniline groups of SAS capturing the peroxy radicals, thereby weakening the chain scission and oxidation of NBR during aging. Another observation, shown in Figure 11, was that both I_C=C_/I_CN_ and I_C=O_/I_CN_ for the NBR/LDH–SAS composites decreased with an increasing content of LDH–SAS, indicating that the thermal oxidative stability of NBR increased with an increasing content of LDH–SAS.

### 3.8. Thermal Properties of NBR Composites

**Figure 12:**
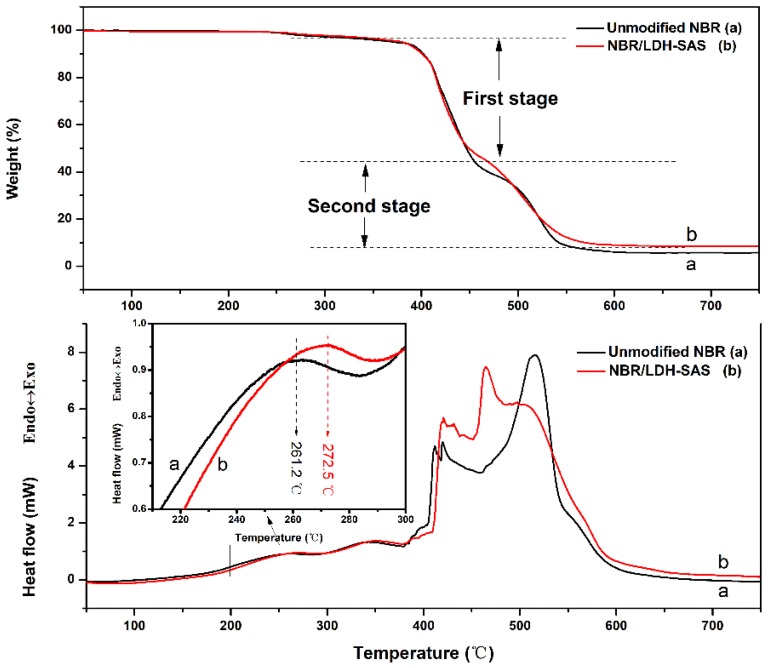
Synchronous DSC and TGA curves of the unmodified NBR and the NBR/LDH–SAS composites.

Thermo-oxidative aging and decomposition of NBR composites were studied by DSC and TGA in an oxygen atmosphere, and the results are shown in Figure 12. The TGA curves of NBR composites showed two obvious decomposition stages. This indicates that the thermo-oxidative degradation process of NBR composites can be reasonably considered to have two stages [64]. The first stage at about 360–450 °C can be considered random scissions of the NBR main chains, producing numerous alkyl radicals. The NBR/LDH–SAS composites showed a slightly lower amount of weight loss in this stage than the unmodified NBR. This indicates that LDH–SAS can effectively retard NBR main chain scission. The second stage at 460–550 °C can be considered further thermo-oxidative decomposition of alkyl radicals [65].

The thermo-oxidative aging peak at 220–300 °C in the DSC curves was due to cross-linking reactions and chain scission [7]. The aging temperature of NBR/LDH–SAS (272.5 °C) was about 11 ± 3 °C higher than that of unmodified NBR (261.2 °C), again indicating that NBR/LDH–SAS has higher thermal stability than unmodified NBR.

### 3.9. Kinetic Analysis of Thermal Oxidative Degradation NBR

The kinetics of thermo-oxidative degradation are important for understanding anti-aging mechanisms [47,66,67] and can illustrate the procedure of thermal oxidative degradation [47].

**Figure 13:**
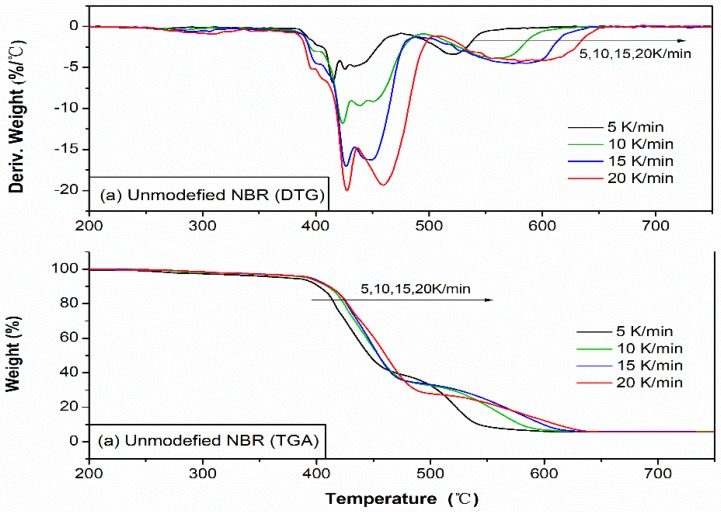

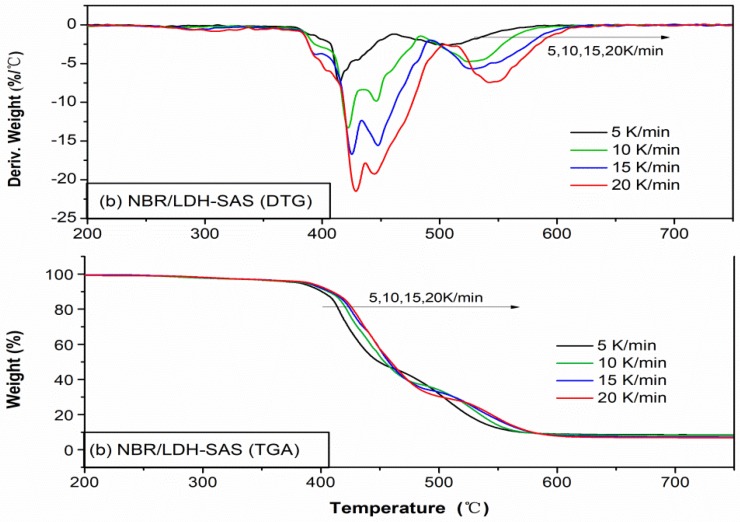
TGA-DTGA thermograms of (**a**) unmodified NBR and (**b**) NBR/LDH–SAS composite at different heating rates.

TGA and DTG curves with different heating rates were recorded to investigate the kinetics of thermo-oxidative degradation of NBR composites, as shown in Figure 13. The peaks in the DTG thermograms shifted to higher temperatures with an increase in the heating rate. The non-isothermal isoconversional Flynn–Wall–Ozawa (FWO) equation is shown as follows [64]:

(6) $$\mathrm{lg}\beta =\mathrm{lg}\;(\frac{\mathrm{AEa}}{\mathrm{RG}(a)})-2.315-0.4567\;\frac{\mathrm{Ea}}{\mathrm{RT}},$$

where β is the TGA heating rate, A is a pre-exponential factor, R is the gas constant, T is the absolute temperature, α is the fractional mass loss, G(α) is a conversional function and Ea is the activation energy of thermal decomposition. With different β, but the same α, G(α) will always be a constant. Therefore, the Ea can be estimated from the slope of the straight line of lgβ against T^−1^ [65].

**Figure 14:**
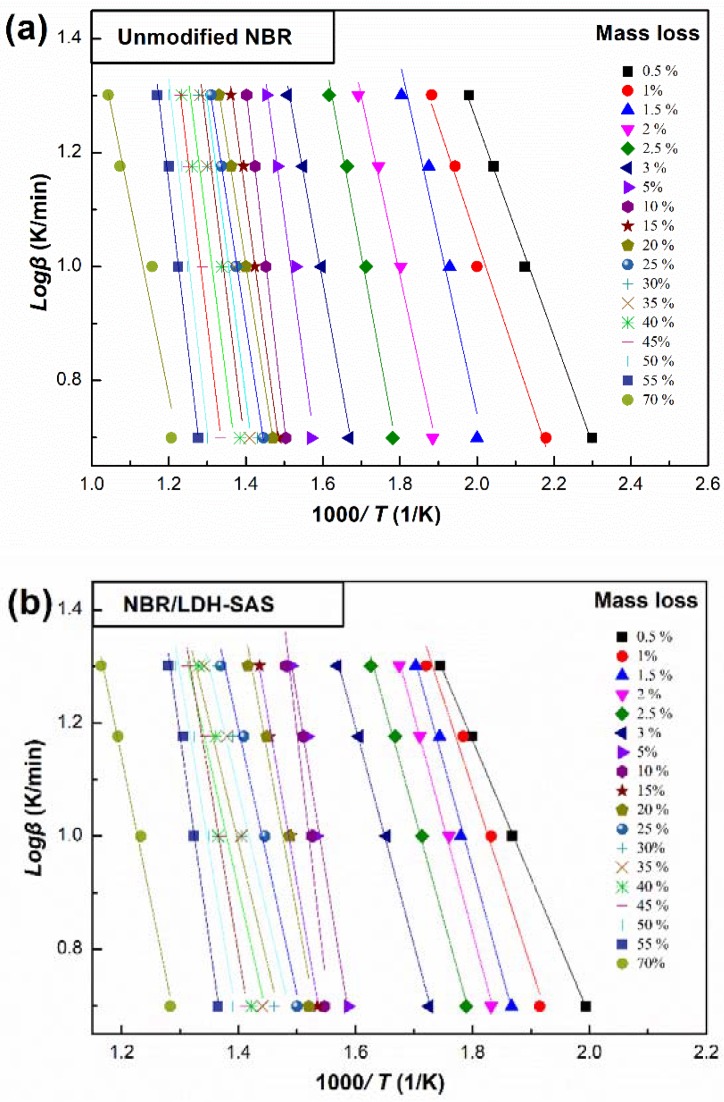
Plots of the logarithm of the heating rate (lgβ) versus the reciprocal of the absolute temperature (1000/T) for the two NBR composites: (**a**) unmodified NBR; (**b**) NBR/LDH–SAS.

**Figure 15:**
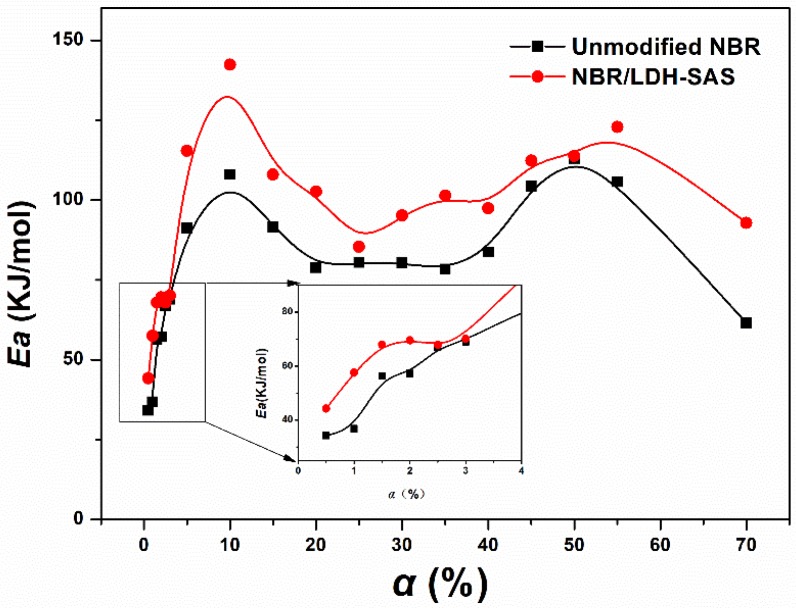
Plots of Ea versus fractional mass loss determined by Flynn–Wall–Ozawa (FWO) analysis.

The relationship between the logarithm of heating rate (lgβ) and the reciprocal of the absolute temperature (1000/T) was linear with coefficients greater than 0.9, as shown in Figure 14. The calculated dependence of Ea on α is further shown in Figure 15. The variation of this dependence can be divided into three stages [62,68,69]. The first stage covers the values of α between 0 and 10%, where Ea increases because of chain initiation [70]. The second stage covers α values between 10% and 20%, where the apparent Ea decreases due to the autocatalytic oxidation reaction [6,68]. The third stage begins slightly above 20% for α. Here, Ea first increases and then decreases with a maximum α equal to 50%. This is caused by the further decomposition of thermo-oxidative intermediate products. It may be noted that the Ea of the NBR/LDH-SAS composites was higher than that of the unmodified NBR in the overall range, because NBR/LDH–SAS had better aging resistance than unmodified NBR, due, as explained above, to radical capture by the aniline groups, restraining degradation, and to the strong LDH–SAS/NBR interactions, which restrain oxygen penetration.

## 4. Conclusions

An organic aniline-containing functional filler, LDH–SAS, was successfully prepared and added to NBR to give NBR/LDH–SAS composites. LDH–SAS was found to be further exfoliated in NBR during processing, which improved the interactions between the filler and the NBR matrix. Calculation of the state equation also suggested a strong interaction between LDH–SAS and NBR, resulting in more physical cross-links. Adding LDH–SAS greatly improves the thermal oxidative aging resistance of NBR composites, because the organic groups of LDH–SAS can capture radicals produced during aging as well as prevent oxygen from penetrating the interior of the rubber matrix. The tensile strength of the NBR/LDH–SAS composites with 5 phr of LDH–SAS decreased by only 0.5% after aging. In contrast, the tensile strength of the unmodified NBR composites decreased by 21% after aging. The kinetics of thermo-oxidative degradation showed that NBR/LDH-SAS had a higher *Ea* than the unmodified NBR during the entire degradation process. This study thus provides an alternative for preventing the frequently occurring thermo-oxidative aging of rubber. The addition of organic SAS to NBR is advantageous in thermo-oxidative aging resistance over other approaches, particularly the double bond approach, because the filler addition is more quantitatively controllable, and thus, property improvement can be predicted, which is practically useful where performance and cost must be balanced.

## Figures and Tables

**Table 1:** Compound formulation of layered double hydroxides (LDH)/ nitrile-butadiene rubber (NBR) composites.

Material	phr ^a^
**NBR**	100
**LDH–NO_3_/LDH–SAS**	0, 1, 3, 5, 7
**ZnO**	5
**Antioxidant D**	1
**Stearic acid**	1
**DM**	0.5
**Sulfur**	1.5
**DOP**	1.5

^a^ Part per hundred rubber.

**Table 2:** Cure time (t_90_) and scorch time (t_10_) of rubber composites.

Compound	Scorch Time/t_10_ (min)	Cure Time/t_90_ (min)
**Unmodified NBR**	10.58	27.87
**NBR/LDH–SAS (1 phr)**	10.57	27.43
**NBR/LDH–SAS (3 phr)**	10.87	27.94
**NBR/LDH–SAS (5 phr)**	11.43	28.46
**NBR/LDH–SAS (7 phr)**	11.96	28.13
**NBR/LDH–NO_3_ (5 phr)**	9.47	27.12

**Table 3:** N_1_ and ΔN_1_ of LDH/NBR composites.

Sample	Unmodified NBR	NBR/LDH–NO_3_ (5 phr)	NBR/LDH–SAS (1 phr)	NBR/LDH–SAS (3 phr)	NBR/LDH–SAS (5 phr)	NBR/LDH–SAS (7 phr)
*Tg* (°C)	−22.1	−20.3	−20.9	−19.9	−19.4	−18.9
E′_AVG_ (MPa)	2.25	3.50	3.00	4.32	4.78	3.89
N_1_ (10^14^)	1.92	2.99	2.56	3.69	4.08	3.32
ΔN_1_ (10^14^)	0	1.07	0.64	1.77	2.16	1.40

N1: the number of network chains per unit volume of rubber; ΔN1: the difference in the number of network chains between NBR/LDH composites and unmodified NBR.
